# Comparison of Outcomes Following Prepectoral and Subpectoral Implants for Breast Reconstruction: Systematic Review and Meta-Analysis

**DOI:** 10.3390/cancers14174223

**Published:** 2022-08-30

**Authors:** Vladimir Mégevand, Matteo Scampa, Helen McEvoy, Daniel F. Kalbermatten, Carlo M. Oranges

**Affiliations:** 1 Department of Plastic Surgery, Guy’s and St Thomas’ NHS Foundation Trust, St Thomas’ Hospital, London SE1 7EH, UK; 2 Department of Plastic, Reconstructive and Aesthetic Surgery, Geneva University Hospitals, University of Geneva, 1205 Geneva, Switzerland

**Keywords:** prepectoral, subpectoral, subcutaneous, breast implant, reconstruction, meta-analysis, comparative, outcomes

## Abstract

**Simple Summary:**

Breast cancer is a growing problem in modern society and is one of the most prevalent cancers among women. Oncologic breast surgery has become an effective therapeutic modality but is often accompanied by major physical and moral impacts on patients. Implant-based reconstruction helps to restore quality of life and aims at providing an optimal esthetic recovery, although the question of implant placement has been too poorly assessed in the past years. In this meta-analysis, we collected and analyzed existing evidence on postoperative outcomes following prepectoral and subpectoral breast reconstruction and allowed us to provide one of the largest pools of patients on this topic. We observed globally higher pain scores following subpectoral implants; however, the rates of postoperative complications remained comparable in a pooled analysis. The risks and benefits of each procedure should be discussed with the patient prior to surgery, and decision making should be guided by surgical expertise.

**Abstract:**

(1) Background: Implant-based breast reconstruction following mastectomy helps to restore quality of life while aiming at providing optimal cosmetic outcomes. Both prepectoral (PP) and subpectoral (SP) breast implants are widely used to fulfill these objectives. It is, however, unclear which approach offers stronger postoperative benefits. (2) Methods: We performed a systematic review of the literature through PubMed, Cochrane Library, and ResearchGate, following the PRISMA guidelines. Quantitative analysis for postoperative pain as the primary outcome was conducted. Secondary outcomes included patient satisfaction and postoperative complications such as seroma, implant loss, skin necrosis, wound infection, and hematoma. (3) Results: Nine articles involving 1119 patients were retrieved. Our results suggested increased postoperative pain after SP implants and significantly higher rates of seroma following PP implants (*p* < 0.05). Patient satisfaction was found to be similar between the two groups; however, the heterogeneity of measurement tools did not allow us to pool these results. The rates of implant loss, skin necrosis, wound infection, and hematoma showed no significant differences between the two cohorts. (4) Conclusion: Our data suggest that both implant placements are safe and effective methods for breast reconstruction following mastectomy. However, homogeneity in outcome measurements would allow one to provide stronger statistical results.

## 1. Introduction

Currently, breast cancer remains a major public health concern, with over 2 million women diagnosed in 2020 and 685,000 deaths reported globally that same year [1]. In this context, implant-based breast reconstruction following mastectomy has been widely described over time and acclaimed for its well-documented restoration of positive self-image and psychological improvement following reconstruction [2,3]. Researchers are constantly evolving new techniques and advances to help to restore the patient’s quality of life while aiming at providing optimal cosmetic outcomes. The popularity of this procedure is essentially driven by its rapid postoperative recovery as well as the lack of donor site morbidity compared with autologous breast reconstruction [4,5]. The selection of the right implant plane, however, remains a controversial decision, and the question of prepectoral (PP) versus subpectoral (SP) planes has been critically assessed by many authors. The traditional SP approach requires a dissection of the pectoralis major and serratus anterior muscles, which act as a protection to the implant and therefore prevent its exposure [6,7]. However, given the demanding manipulation of the muscle, it is often associated with increased postoperative pain and shoulder dysfunction. The PP, or subcutaneous approach, has more recently emerged as an alternative to the SP implant placement, avoiding disruption of the muscle and therefore offering better immediate postoperative functional results, although evidence in the literature suggests higher risks of mastectomy skin flap necrosis, implant extrusion, and capsular contracture [8,9]. When the PP technique is performed, the implant is usually covered with an acellular dermal matrix (ADM), a sterile tissue matrix from which the cells have been devitalized. The ADM acts like a scaffold for new tissue formation and allows rapid vascular ingrowth. It replaces implant coverage by the pectoralis major and seems to be associated with reduced postoperative pain and improved cosmetic outcome, offering a more natural ptosis and reduced incidence of capsular contracture [7,9].

Several prospective and retrospective studies have reported specific outcomes resulting from PP versus SP implants in a comparative view, such as pain scores, quality of life, and postoperative complications. With this systematic review and meta-analysis, we aimed to summarize the current evidence on both techniques and to evaluate the spectrum of reported outcomes associated with PP and SP breast reconstruction in a comparative view.

## 2. Materials and Methods

The study was conducted in accordance with the Preferred Reporting Items for Systematic reviews and Meta-analyses (PRISMA) [10]. The protocol has not been registered

### 2.1. Literature Search Methodology

An exhaustive literature search was performed through PubMed, Cochrane Library, and ResearchGate, aiming at all the studies on breast reconstruction comparing outcomes following PP and SP implants. The keywords prepectoral, subpectoral, subcutaneous, implant-based reconstruction, and breast implants were used as search strings. Inclusion and exclusion criteria were defined using PICOS (Table 1) before conducting the systematic review.

### 2.2. Selection Process

The titles and abstracts were independently scrutinized by two authors (V.M. and M.S.) to identify relevant articles for this review, using Rayyan software (Cambridge, MA, United States of America for systematic reviews [11]. Any disagreement between reviewers was resolved by consensus after a consultation with a third independent reviewer (C.M.O.).

Full-text articles with patients receiving PP or SP implants following mastectomy for treatment or prevention of breast cancer were included and fully read. Non-English articles, meta-analyses, reviews, case reports, letters to the editor, isolated abstracts, and non-oncologic-based breast augmentations were excluded. No limitations were applied to the age or ethnicity of the patients, or to the type of implant used for breast reconstruction. In addition, the reference lists of all relevant articles were scrutinized to identify additional relevant studies.

### 2.3. Data Extraction

Data from eligible studies were extracted by two authors (V.M. and M.S.) using a standardized Excel (Microsoft Corporation, Redmond, Washington, United States of America) file after reviewing each publication. The following data were collected: study characteristics (first author, publication year, country of origin, study type, the total number of patients and number of patients in each cohort, mean age, BMI, and median follow-up), type of procedure (plane of implant placement and one- or two-staged procedure) and clinical outcomes (pain scores, patient satisfaction, and postoperative complications). No attempt to retrieve missing data from the authors of the included papers was made.

### 2.4. Outcome Assessment

Postoperative outcomes were assessed and compared for each type of breast reconstruction. All outcomes obtained from the selected studies are reported with the same measurements retrieved from the articles and compared if homogeneous. Outcomes reported in three or more studies and assessed with a comparable tool were included in the meta-analysis. Postoperative pain had to be reported in every included article. Secondary outcomes included patient satisfaction as well as postoperative complications such as seroma, implant loss, skin necrosis, wound infection, and hematoma.

### 2.5. Statistical Analysis

The Mantel-Haenszel method was used to combine results across studies [12]. Outcome comparisons are expressed as risk ratio (RR) for postoperative complications and as mean difference (MD) for continuous variables such as postoperative pain, both expressed with a 95% confidence interval (95% CI). A random-effects model was used for all outcomes, given that both intrinsic and extrinsic variabilities between the studies were expected to be high in terms of study population and outcomes assessment. Statistical heterogeneity was tested using chi-square and inconsistency (I^2^) statistics. I^2^ values below 30% represent low heterogeneity, values between 30% and 50% represent moderate heterogeneity, values between 50% and 70% represent substantial heterogeneity, and values over 70% represent high heterogeneity between the studies [13]. Results were considered statistically significant when the *p*-value was inferior to 0.05 (*p* < 0.05). When standard deviation was not reported, we used the interquartile ratio (IQR) and calculated the ratio IQR/1.3.

## 3. Results

**Figure 1:**
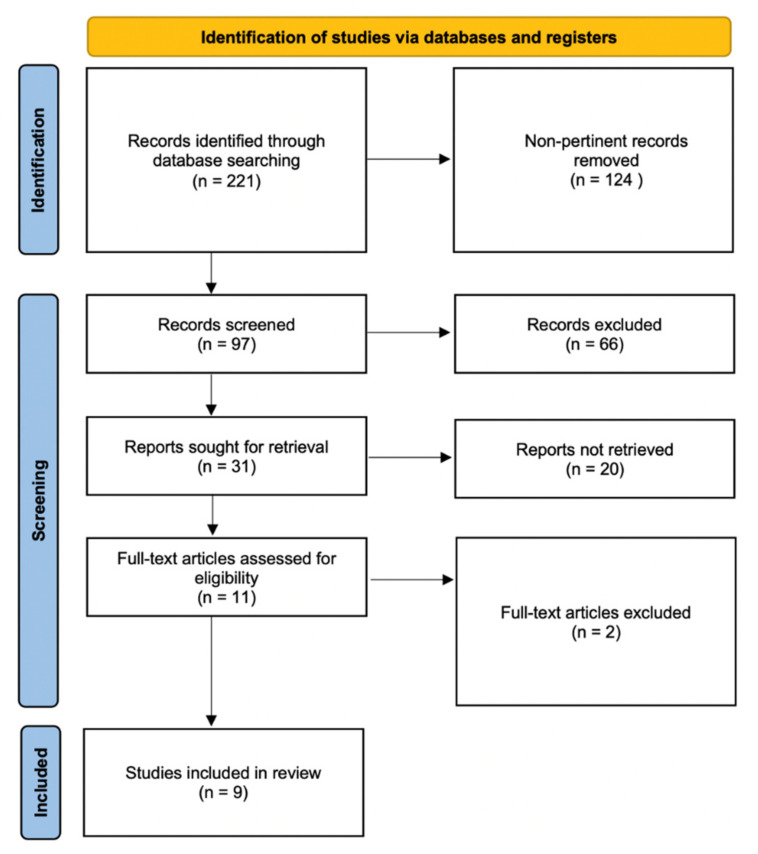
Literature search flowchart.

The literature search yielded nine relevant articles [14,15,16,17,18,19,20,21,22] and sources of information on outcomes following PP versus SP implants for breast reconstruction following mastectomy (Table 2). The literature search flowchart is displayed in Figure 1.

All studies were comparative, three of them being prospective [3,4,9] and six being of retrospective nature [16,17,18,19,21,22]. The studies included a total of 1119 patients, corresponding to 502 (45%) PP and 617 (55%) SP breast implants. The size of the included studies ranged from 34 to 238 patients, with a follow-up between 1 month and 4 years after surgery. Nelson et al. [22] initially included 921 patients and then performed a matched cohort study. Therefore, outcomes were reported on 238 patients after matching and assessing the distribution propensity scores between patients. All studies were published between 2018 and 2022, covering a study period from 2016 to 2021, except from Walia et al. [16], whose study period started in 2011. All studies were performed as single-center studies and included adult female patients who underwent immediate breast reconstruction following mastectomy either with definitive implants or with tissue expanders. The type of implants varied from one study to another, but ultimately the outcomes were always compared between PP and SP breast implants. The population age and BMI were homogenous between the studies. Three studies [18,19,22] included patients undergoing nipple-sparing mastectomies only, one study [20] included patients undergoing skin-sparing mastectomies only, two studies [15,17] included both types, and three studies [14,16,21] did not specify the type of mastectomy undergone by the included patients. In almost all of the included studies, most patients did not receive radiotherapy, and in one study [14], no patient received any.

**Figure 2:**
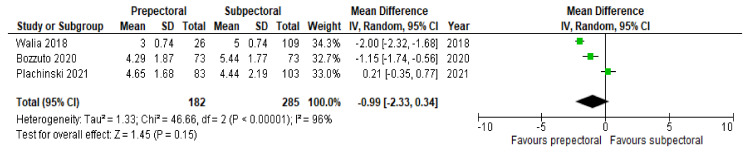
Postoperative pain [16,18,21].

Postoperative pain was assessed in all the included studies, and most of them (seven studies, 78%) suggested significantly higher rates of postoperative pain in the SP cohort. However, due to a lack of standardized measurements, we were not able to perform a meta-analysis of all studies. In our meta-analysis, we included the results of Walia et al. [16], Bozzuto et al. [18], and Plachinski et al. [21], all three using the VAS. There was a strong heterogeneity across the studies (*p* < 0.00001, I^2^ = 96%). The statistical analysis using the random-effects model showed comparable rates of postoperative pain between the two cohorts (MD, −0.99; 95% IC, −2.33–0.34; *p* = 0.15; Figure 2).

Patient quality of life after surgery was assessed in six studies. Cattelani et al. [15] and Franceschini et al. [19] suggested significantly higher satisfaction rates following PP surgery. In contrast, Baker et al. [14], Walia et al. [16], Lee et al. [20], and Nelson et al. [22] found no statistically significant difference between the two cohorts. Given the heterogeneity of measurement scales and insufficient information, inclusion, and comparison of studies in the meta-analysis were impossible.

**Figure 3:**
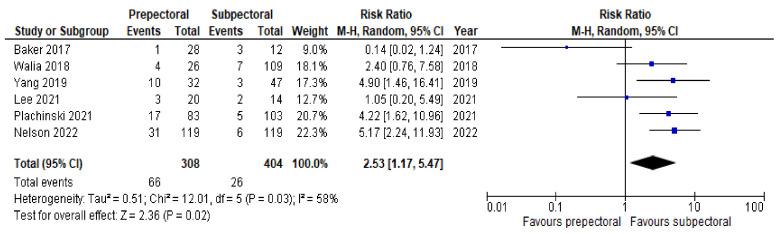
Seroma [14,16,17,20,21,22].

**Figure 4:**
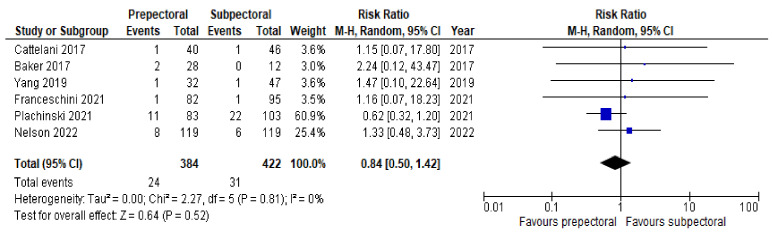
Implant loss [14,15,17,19,21,22].

**Figure 5:**
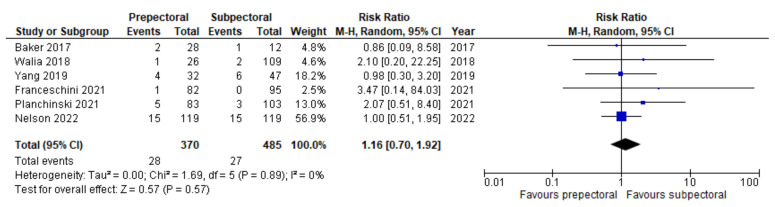
Skin necrosis [14,16,17,19,21,22].

**Figure 6:**
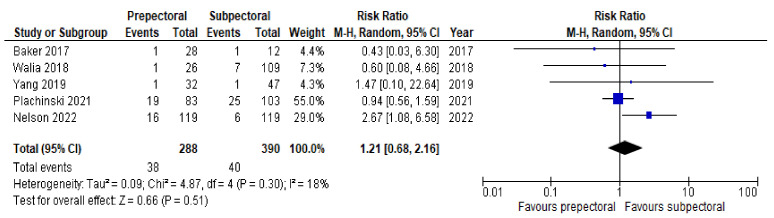
Wound infection [14,16,17,21,22].

**Figure 7:**
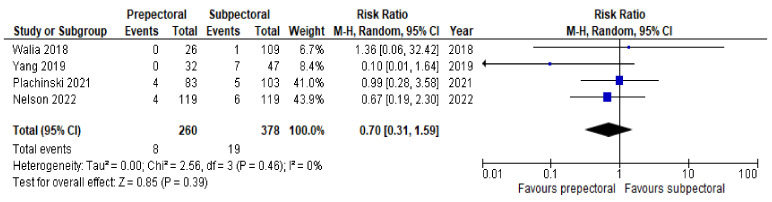
Hematoma [16,17,21,22].

Regarding postoperative complications, some studies reported the outcomes on a per-patient basis regardless of breast laterality, and others reported them according to the individual breast. However, the results remained comparable as a whole and allowed us to perform a pooled analysis. Six studies [14,16,17,20,21,22] (67%) were pooled to compare the rates of seroma between the PP group and the SP group. Substantial heterogeneity was observed, with an I^2^ value of 58%. The results showed significantly higher rates of seroma in the PP group (RR = 2.53; 95% IC, 1.17 – 5.47; *p* = 0.03; Figure 3). Six studies [14,15,17,19,21,22] (67%) were pooled to compare the rates of implant loss between the PP and SP groups. Low heterogeneity was observed between the studies (I^2^ = 0%; *p* = 0.81), and the rates of implant loss were comparable between PP and SP (RR = 0.84; 95% IC, 0.50–1.42; *p* = 0.57; Figure 4). Six studies [14,16,17,19,21,22] (67%) were pooled to compare the rates of skin necrosis between the two cohorts. The results showed no significant difference between these groups (RR = 1.16; 95% IC, 0.70–1.92; *p* = 0.57; Figure 5) with low heterogeneity observed between the studies (I^2^ = 0%; *p* = 0.89). Five studies [14,16,17,21,22] (56%) were pooled to compare the rates of wound infection between the PP group and the SP group. A low heterogeneity was observed in this study pool, with an I^2^ value of 18% (*p* = 0.30), and the results showed no significant difference in wound infection between the two cohorts (RR = 1.21; 95% IC, 0.68–2.16; *p* = 0.51; Figure 6). Lastly, four studies [16,17,21,22] (44%) were pooled to compare the rates of hematoma between the PP group and the SP group. Low heterogeneity was observed (I^2^ = 0%; *p* = 0.46), and the results showed no significant difference in hematoma between these two groups (RR = 0.79; 95% IC, 0.31–1.59; *p* = 0.39; Figure 7).

## 4. Discussion

In the present study, we collected and analyzed existing evidence on postoperative outcomes following PP versus SP breast reconstruction. Our findings show that PP and SP breast reconstructions are similarly effective in restoring quality of life after mastectomy and show a poor difference in terms of postoperative complications. Yet, the lack of homogeneity in outcome measurements between the studies limits the pertinence of our results.

One of the major advantages of PP over SP implant placement is the reported difference in postoperative pain, mainly due to the absence of chest wall musculature manipulation. This hypothesis is supported by emerging data on pain scores associated with tissue expanders, with results trending toward less pain after placement of PP implants [6,14,16,22]. Two measurements allow for the evaluation of pain after surgery: the first one is a pain assessment scale or questionnaire given to the patient postoperatively, and the second one is the amount of opioid use after surgery. Four studies [16,18,20,21] used the visual analogue scale (VAS). One study [14] used the Likert scale, one study [15] used the Brief Pain Inventory, one study [17] used the numeric rating scale, one study [19] used the QOL assessment PRO, and one study [22] used a one-to-four scale to assess postoperative pain. To achieve uniformity, only those studies using the VAS were pooled to compare the rates of postoperative pain. However, we decided to exclude the study of Lee et al. [20] from our meta-analysis as they used the VAS on a long time scale and because they assessed postoperative pain based on the range of motion of the shoulder and not at rest. Our data showed lower postoperative pain scores following PP implants in seven (78%) of the included studies [15,16,17,18,19,20,22] based on pain questionnaires, and pooled analysis combining the results of three studies [16,18,21] showed no significant difference between the two groups. There was a strong heterogeneity across the studies, and this can be explained by the fact that the study populations and the postoperative analgesic protocols differed among the studies. Baker et al. [14] found no significant difference in cumulative pain scores between PP and SP implants over the immediate postoperative period or at any postoperative time point. They hypothesized that the majority of pain arises from the mastectomy and related axillary procedures and that elevation of the chest wall musculature only accounts for a minor part of the perceived pain. Yet, previous studies have indicated that the incidence of pain after cancer surgery is significantly higher when breast reconstruction is performed, especially following the SP approach, with 49% of patients experiencing upper body acute and chronic pain syndrome [23,24]. Plachinski et al. [21] found similar results, with no significant difference in pain scores between the two cohorts and that the evaluation of pain should be more inclusive of other intrinsic criteria such as chronic pain, comorbidities, age, and BMI, in opposition to most of the previous studies [15,25,26]. Lee et al. [20] observed a significant difference in favor of the PP group when pain was assessed during shoulder motion two weeks postoperatively, and, interestingly, the scores of shoulder disability were significantly higher in the SP group. In light of these results, there is strong evidence that muscle sparing using the PP approach prevents increased postoperative pain. However, the lack of baseline comparison between all the included studies limits our findings, and further research using standardized pain assessment tools should be performed to reinforce these data. The findings of reduced postoperative pain are aligned with those of significantly reduced opioid consumption after surgery in PP patients, as suggested by Cattelani et al. [15] and Bozzuto et al. [18], leading to shorter hospital length of stay and faster return to work. The mortality rates of opioid overdose have multiplied by three in the last two decades. One major contributor to opioid misuse is physician over-prescription, and it was proved that each week of opioid use increases the risk of long-term dependence [27]. Copeland-Halperin et al. [25], in their study comparing prepectoral versus dual-plane breast reconstruction, observed that the PP group required 33% fewer days on opioid medication and were 66% less likely to require prescription refills; hence, the important role of the surgeon to avoid additional risk by postoperatively decreasing the amount of opioid prescription. However, no dual-plane patients were included in our study, given the potential biases related to complications arising from the uncovered part of the implant, potentially influencing our results. Bozzuto et al. [18] and Walia et al. [16] both observed significantly shorter hospital lengths of stay in the PP group compared with the SP group. These results confirmed the findings of previous studies showing earlier discharge in PP patients with an average length of stay of 0.6 days compared with 2 to 3 days for SP patients [28,29]. Additionally, recent evidence demonstrated that earlier discharge was associated with increased patient satisfaction and psychological well-being [30].

Patient satisfaction and quality of life can be estimated by means of a questionnaire given after surgery. Baker et al. [14], Cattelani et al. [15], and Nelson et al. [22] used the Breast-Q score, which is a validated patient-reported outcome instrument designed to evaluate outcomes among women undergoing different types of breast surgery [31]. Walia et al. [16] used the same tool but reported the Breast-Q score for each item individually and not overall. Of the two remaining studies that evaluated the patient’s quality of life, Franceschini et al. [19] used the QOL assessment PRO, and Lee et al. [20] used the SF-36 score. Of those who employed the Breast-Q, Baker et al. [14] used the full Breast-Q questionnaire, including the Satisfaction with Breast scale, the Satisfaction with Outcome scale, the Psychological Well-Being scale, the Physical Well-Being scale, and the Sexual Well-Being scale. Cattelani et al. [15] used the Psychosocial Well-Being scale and the Aesthetic Satisfaction scale. Nelson et al. [22] used the Physical Well-Being scale only. Achieving uniformity between those three studies was not possible given the lack of baseline comparison; therefore, their results were not pooled. There is evidence that the avoidance of manipulation of the chest wall musculature with the PP approach offers better results in terms of quality of life [6,22,23,24]. Yet, one should remain critical when looking at these results, and further research should be performed on this matter to confirm or disprove these data.

Several researchers have compared the rates of postoperative seroma, implant loss, skin necrosis, wound infection, and hematoma between PP and SP implants. Our results suggested a significant difference in terms of seroma in favor of the PP group, but no differences were noted when assessing the other complications. Anatomically, SP implants are supported by the pectoralis major muscle, therefore allowing the mastectomy skin flap to tightly adhere to the underlying soft tissue [21]. PP implants are positioned in such a way that they create a barrier between these two planes; therefore, by means of an artificial dead space, they allow seroma to more easily accumulate. Plachinski et al. [21] dismissed a causal relationship between seroma rates and patient BMI, mastectomy weight, and ADM usage, reinforcing the hypothesis that seroma formation may be strongly related to implant positioning. Nahabedian [32], in his study on prepectoral breast reconstruction, suggested an association between flap thickness and tissue perfusion, which he determined using fluorescent angiography. We may hypothesize that when skin perfusion is critical, SP implant placement should be considered. In our study, implant loss was reported as any unexpected removal of an implant described by each author following wound infection [14,19], skin necrosis [14,15], or any other complications [17,21,22]. When assessing postoperative infection in their study, Yang et al. [17] included both early (<90 days) and late (>90 days) infections. Given the purpose of our study to evaluate immediate postoperative complications, we did not consider their case of late infection in our results. 

A recently published meta-analysis by Li et al. [33] comparing PP and SP breast implants after mastectomy obtained similar results to ours in terms of skin necrosis and implant loss and further suggested a reduced risk of capsular contracture following PP implants. However, one major limitation of their study is that they did not give specific details on partial and total SP implant placement, which are different surgeries with o different clinical outcomes. Although there is no significant difference in postoperative complications or quality of life, the difference in operative techniques may lead to different pain scores given the variable extent of muscle dissection.

The results of this meta-analysis should be considered with caution due to the number of limitations and potential biases influencing these findings. We collected information extracted from comparative studies evaluating postoperative outcomes following PP and SP breast reconstruction after mastectomy. The first limitation of our findings is the lack of homogeneity in measurements of postoperative pain and patient satisfaction between the studies. To minimize heterogeneity and reduce bias in our final pooled analyses, we only included studies using the same tool for each outcome assessment, therefore excluding the outcomes from other relevant studies. Furthermore, no differentiations were made between one- (definitive implant) and two-staged (tissue expander) surgeries, nor on the use of ADM or mesh. We think that these are important criteria to consider, especially in terms of wound infection when implanting additional materials such as ADM and mesh or when requiring multiple secondary procedures from the practitioner after the initial surgery, as in the case of tissue expanders. Moreover, when performing an exhaustive literature search, we considered that not all studies specified the kind of subpectoral approach used for reconstruction (fully subpectoral or dual-plane). In order to avoid any bias in pooled analysis and, therefore, in the interpretation of our results, we limited our data to those studies focusing on either PP or fully SP reconstructions. Indeed, the dual-plane approach can potentially lead to complications related to the uncovered part of the implant, although this approach surgically consists of a dissection of the pectoralis major, which is synonymous with SP. Lastly, this study included a small number of publications and, particularly, a very limited number of prospective studies.

Overall, surgical outcomes appear to be similar with both PP and SP implants. Despite the nonstatistically significant differences, some outcomes assessed in this study tend to favor PP implants, whereas others favor SP implants. SP implants offer the advantage of reducing the risk of seroma, exposure to lower risks of skin flap necrosis, and wound infection. However, some plastic surgeons seem to prefer the use of PP implants given the less extensive functional impairment and, therefore, lower pain scores associated as well as the reduced rates of hematoma and implant loss.

## 5. Conclusions

Our data suggest that both PP and SP implant placements are safe and effective approaches to breast reconstruction following mastectomy, although SP implants tend to offer significantly lower rates of seroma. Most authors agree that PP implants provide lower pain scores. No differences were noted in terms of quality of life, implant loss, skin necrosis, wound infection, or hematoma. Yet, a stronger baseline comparison in outcome measurements should guide further research into more comprehensive pooled analyses and would allow us to reinforce these data. Both reconstructive options should be included in the armamentarium of the practitioner, with the decision for implant placement being based on individual needs and guided by surgical expertise.

## Figures and Tables

**Table 1:** PICOS for inclusion and exclusion criteria.

	Inclusion	Exclusion
Population	Adult patients who underwent IBR following mastectomy for treatment or prevention of breast cancer	Non-oncologic-related breast augmentations
Intervention	Prepectoral reconstruction with or without ADM	Non-implant-based breast augmentations
Comparator	Subpectoral reconstruction	Dual-plane reconstruction
Outcomes	Primary outcome: pain Secondary outcomes: patient satisfaction, seroma, implant loss, skin necrosis, wound infection, hematoma	Primary outcome not assessed in study
Study design	Prospective and retrospective comparative studies	Non-English articles, meta-analyses, reviews, case reports, letters to the editor, isolated abstracts

IBR = implant-based reconstruction; ADM = acellular dermal matrix.

**Table 2:** Characteristics of the included studies.

Author	Year	Total Patients	PP	SP	Mean Age PP (y)	Mean Age SP (y)	Mean BMI PP	Mean BMI SP	Follow-Up
Baker et al. [14]	2018	40	28	12	47.5	48	26.0	23.4	***
Cattelani et al. [15]	2018	84	39	45	52.9	52.3	24.9	26.1	12 mo
Walia et al. [16]	2018	135	26	109	51.4	48.6	24.3	26.1	30–60 d
Yang et al. [17]	2019	79	32	47	48.9	46.4	23.5	21.3	52 mo
Bozzuto et al. [18]	2020	146	73	73	49	49	23.7	23.2	***
Franceschini et al. [19]	2021	177	82	95	47	44	23.9	24.8	18 mo
Lee et al. [20]	2021	34	20	14	46.2	46.8	20.9	21.3	***
Plachinski et al. [21]	2021	186	83	103	47.9	49.9	28.1	26.1	19 +/− 11 mo
Nelson et al. [22]	2022	238	119	119	50.3	48	26.4	26.2	***

PP = prepectoral; SP = subpectoral; BMI = body-mass index (kg/m^2^); y = years; mo = months; d = days; *** = data not reported.
